# A data centric HitL framework for conducting a systematic error analysis of NLP datasets using explainable AI

**DOI:** 10.1038/s41598-025-13452-y

**Published:** 2025-08-19

**Authors:** Ahmed El-Sayed, Aly Nasr, Youssef Mohamed, Ahmed Alaaeldin, Mohab Ali, Omar Salah, Abdullatif Khalid, Shaimaa Lazem

**Affiliations:** 1 Computer and Systems Engineering Department, Faculty of Engineering, Alexandria University, Alexandria, Egypt; 2 Informatics Research Institute, City of Scientific Research and Technological Applications, New Borg El-Arab, Egypt

**Keywords:** Arabic emotion analysis, Explainable AI, XAI, Machine learning, LIME, SHAP, Human centered AI, Human in the loop, Error analysis, Computer science, Information technology

## Abstract

The interest in data-centric AI has been recently growing. As opposed to model-centric AI, data-centric approaches aim at iteratively and systematically improving the data throughout the model life cycle rather than in a single pre-processing step. The merits of such an approach have not been fully explored on NLP datasets. Particular interest lies in how error analysis, a crucial step in data-centric AI, manifests itself in NLP. X-Deep, a Human-in-the-Loop framework designed to debug an NLP dataset using Explainable AI techniques, is proposed to uncover data problems related to a certain task. Our case study addresses emotion detection in Arabic text. Using the framework, a thorough analysis that leveraged two Explainable AI techniques LIME and SHAP, was conducted of misclassified instances for four classifiers: Naive Bayes, Logistic Regression, GRU, and MARBERT. The systematic process has resulted in identifying spurious correlation, bias patterns, and other anomaly patterns in the dataset. Appropriate mitigation strategies are suggested for an informed and improved data augmentation plan for performing emotion detection tasks on this dataset.

## Introduction

Conventional AI model development prioritizes architecture design followed by optimization of hyperparameters. Advancements often involve new or enhanced architectures, with data preparation (pre-processing and augmentation) as the first and only data manipulation step. This approach assumes inherent data quality, allowing researchers to focus on tuning and improving the model as the primary variable while treating the data as constant. Model quality is assessed using accuracy and similar metrics. Low accuracy may prompt suggestions for training additional data or the exploration of more complex models. However, even with improved accuracy, the quality of the model remains questionable if the underlying data is flawed. It could be additionally misleading if the data is never scrutinized resulting in cases of rightly classified data for the wrong reasons, for instance. In these scenarios, observed accuracy gains may not reflect genuine improvement.

Data collection and annotation are expensive endeavors. The assumption underlying the conventional approach, often relying on large, perfectly labeled datasets, proves unrealistic in practice. Noise and inherent biases, which often go undetected, further complicate matters. While techniques such as data augmentation, semi-supervised learning, transfer learning, and active learning offer valuable cost-reduction strategies by automating or bypassing those expensive endeavors, further exploration of data optimization techniques is warranted. This is where the data-centric approach comes into play^1–3^. Data-centric AI (DCAI) draws attention to the central role data plays in elevating AI model performances. It is motivated by the increased availability and democratization of optimized AI architectures and models, allowing the research community to shift their focus and bandwidth to data research^2^. The key to DCAI is treating data as a first class citizen in the discussion on using AI in real-world applications^4^. Further, DCAI treats data as dynamic, where data refining using error analysis, augmentation, and quality assessment becomes an integrated process into the model development life cycle^2^. The major problems plaguing data such as noisy data, dirty data, poisoned data, missing or incorrect features and labels, bias and unfairness are at the center of DCAI scholarship^1^. Many of these techniques have already existed before the term was coined^2,5^ but as a single step rather than embedded within a process for improving both the data and the model together. Zha et al.^3^ provided a general overview of the data-centric AI process, the need to use it instead of the model-centric approaches, and its’ steps - Training data development, Inference data development, and Data maintenance - to systematically engineer high quality data for building AI models. The discourse on DCAI is still growing and therefore has been abstract in nature with limited specificity on how the approach would work with various kinds of messy real-world datasets. Further, techniques more specific to AI such as Explainable AI (XAI) have not been thoroughly examined in DCAI discussions.

This research fills this gap by proposing a DCAI inspired framework for error analysis and data refinement in the domain of Arabic NLP^6^, using XAI techniques. The chosen case study is the problem of emotion detection^7^. Quality emotion detection from text is the backbone for enabling a wide class of applications, most notably chatbots that could be empathic in conversation with users^8^. Emotion detection in Arabic poses DCAI specific challenges and opportunities. Despite its popularity on the Internet, Arabic language datasets’ availability is low compared to other popular languages such as English. Arabic NLP developers often start building their models using the available minimum viable data^9^. This research demonstrates that a DCAI approach could help refine and decide on informed data augmentation efforts. Further, Arabic datasets collected from social media lack proper knowledge about the context of the data, e.g., collecting tweets using hashtags. This, in turn, makes data annotation subject to inconsistencies and unpredictable biases. The emotion detection task is even more challenging due to the inherent subjectivity in interpreting emotions. A DCAI would help developers improve data quality by unveiling inconsistencies in data annotation and potential sources of bias.

In particular, X-Deep, a Human-in-the-Loop (HitL) framework, is contributed to leverage XAI for refining and debugging NLP datasets. The framework was used to systematically analyze misclassified instances across four classifiers and examine them using two XAI techniques. This process has resulted in the identification of patterns that can improve dataset quality. To the best of our knowledge, neither the identified patterns nor the process used to uncover them has been addressed in the literature. The practical insights from this research contribute to broadening and advancing the DCAI discourse on NLP data.

The rest of the paper is organized as follows. A summary of the related work is presented in Section “Related work”. The X-Deep framework is proposed in Section “Approach”. The results are presented in Section “Experimental results”, and discussed in Section “Discussion”. The paper is concluded and recommendations for future work are outlined in Section “Conclusion and future work”.

## Related Work

This section reviews the existing research efforts for developing XAI debugging frameworks and Arabic emotion detection models.

### XAI Debugging Frameworks

The use of XAI has exploded in popularity in recent years^10^ as means to understand the inner working of black-box models and justify their decisions^11^. The provided explanations have various uses, whether in supporting human decision making, verifying model reasoning, finding potential causes of errors, or simply building trust in models^12^. This is particularly important with the growing concern regarding trustworthy AI and responsible AI as AI takes on increasingly critical roles in our lives^13^ such as in medical or safety-critical systems.

While XAI has shown promise in improving models, its application in debugging often focused on internal model workings rather than data quality^14^. Critics argue that current XAI methods excel at explaining simple, non-realistic scenarios^15^. They may overlook spurious correlations or lack a systematic approach to identify data-related issues, leading to the exploration of only anticipated data bugs^14^. In the field of NLP, involving human users in debugging using XAI has proven to be beneficial. Hartmann et al.^16^ showed how training a model with access to human explanations can improve data efficiency and model performance. Jinghui et al.^17^ aimed to increase the generalization of models in few-shot learning scenarios using human-identified rationales in the training process. This approach boosted the performance of models in both in-distribution and out-of-distribution datasets and made models more robust. Mosca et al.^18^ argued that interpretability and human participation are the fundamentals of complex NLP models and proposed a framework for real-time explanation-based interaction with NLP models. Through this framework, users provided feedback to the model’s predictions and explanations, and that feedback is then used to fine-tune the model. This approach helped in reducing bias with minimal impact on model performance. Dong-Ho et al.^19^ also proposed a framework for explanation-based model debugging using user feedback on task-level and instance-level explanations to guide the model to make predictions following the correct reasons. This approach led to significant improvement on model generalizability. Notably, most of the debugging frameworks explored hate-speech detection, a task where the source of potential bias can be predicted. Our work expand these efforts to emotion detection tasks, where potential sources of bias(es) or spurious correlations are challenging to predict beforehand.

### Emotion Detection in Arabic NLP

Arabic’s unique linguistic features present significant complexity, especially in emotion analysis and during pre-processing due to its rich morphology, dialectical variations, cultural nuances, and diacritics and character variation. Previous research has largely focused on increasing model size in Arabic NLP, neglecting the potential benefits of customized, task-specific pre-processing techniques^20^. The work on emotion detection from Arabic text is rather scarce, and the available annotated datasets are limited. Semi-supervised self-learning and transfer learning had shown promising results as alternative approaches^21^. Abdelwahab et al.^22^ analyzed sentiment in Arabic tweets about LASIK eye surgery using LSTMs and LIME^23^. Interestingly, skipping common pre-processing steps improved their accuracy to 79.1%, suggesting a trade-off between simplicity and performance in this specific case. Aljameel et al.^24^ built a sentiment analysis model to assess public awareness of COVID-19 precautions in Saudi Arabia, aiming to inform public health responses. Analyzing Twitter data during quarantine, they found the Naive Bayes model performed best (80% accuracy) and identified the southern region as most aware, while the central region showed lower awareness, aligning with confirmed COVID-19 cases. Abdelwahab et al.^25^ addressed the challenge of multi-dialect Arabic sentiment analysis using CNN, LSTM, CNN-LSTM, and RNN models on word and character levels. They found that LSTMs perform best at word-level (79% accuracy) and CNNs at character-level (86%). Larger and more diverse datasets significantly improve performance compared to single-dialect datasets. Annotating large datasets for a comprehensive task such as emotion analysis is challenging^26^. A DCAI approach could be helpful in refining the annotation process for smaller datasets by unveiling potential sources of data problems before expanding the annotation efforts.

Analyzing the literature shows that existing research heavily employs XAI for model interpretation or fine-tuning. Data-centric approaches, on the other hand, often overlook the potential of XAI. This work seeks to bridge this gap by leveraging XAI within a data-centric framework. The inherent complexities of Arabic language make it an ideal case study to demonstrate the value of the proposed approach.

## Approach

While previous studies have used XAI tools for model debugging or post-hoc interpretation, they often treat explainability as an end rather than a means for improving the model development process. In contrast, this study introduces a novel framework that integrates HitL feedback and XAI insights to inform key stages of model development, including data pre-processing, model selection, and evaluation. Specifically, the proposed framework, X-Deep expands the use of XAI tools to perform the following.

- Identify anomalies and spurious correlations in the data that may lead to biased predictions.
- Compare the behavior of different model architectures to uncover strengths and weaknesses with respect to a specific dataset.
- Refine training data and guide model selection based on interpretable signals.
- Go beyond surface-level accuracy by ensuring that model predictions are justified by meaningful patterns in the data, in other words, they are right for the right reasons.

This comprehensive approach shows how explainability can inform and improve each stage of development, from data curation to model evaluation. We demonstrate the utility of this framework through a case study in Arabic emotion detection, a challenging task where model bias, language complexity, and limited resources make interpretability especially valuable. The ultimate objective is not solely to achieve high accuracy, but rather to improve data collection in order to develop a model that provides explainable and coherent outputs.

X-Deep, a HitL framework, to help NLP developers in analyzing datasets to detect recurring patterns and ambiguities, that we will call anomalies, which may mislead classifiers. Rather than relying solely on standard accuracy metrics, X-Deep uncovers deeper quality issues within the data. Highlighting these anomalies offers insights for pre-processing or additional data collection, avoiding brute-force methods like indiscriminate data collection or uniform pre-processing. The goal is to augment human analysis with XAI to identify anomalies^2^, where the augmentation of human expertise was shown to reveal latent weaknesses and refine critical processes such as data acquisition and pre-processing^12^, ^27^, ^28^.

**Fig. 1:**
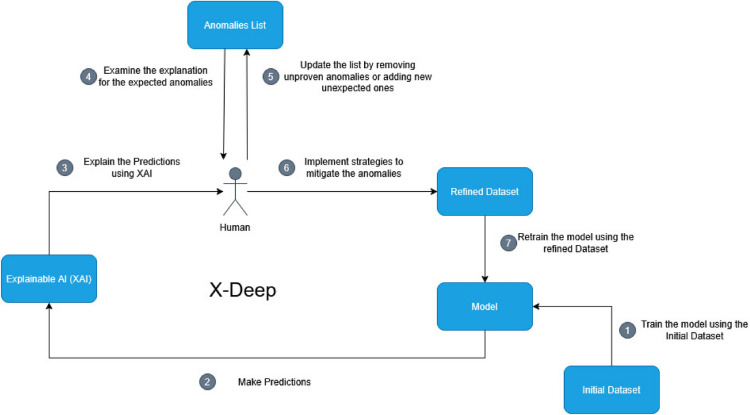
X-Deep HitL framework illustration.

As shown in Fig. 1, the framework begins by training models on a dataset and identifying misclassifications, using XAI to inspect and interpret these misclassifications to infer underlying data anomalies. The process can also extend to correctly classified samples suspected of flawed reasoning. We define anomaly patterns as recurring phenomena that lead to systematic misclassifications across classifiers, indicating inherent data issues such as incorrect labels, biases, or spurious correlations–problems that cannot be resolved by blindly adding more data. XAI tools are deployed to confirm these anomalies are systemic (not isolated instances) and to validate proposed mitigation strategies.

Four diverse classifiers, Naive Bayes, Logistic Regression, GRU, and MARBERT, along with two model-agnostic XAI methods, LIME^23^ and SHAP^29^, were used in the presented experiments to prevent technique-specific or classifier-specific biases in anomaly detection. An initial list of hypothesized anomalies helped initiate and guide the iterative process of anomaly detection. The list was formed based on an exploratory analysis of the dataset, including label distributions, frequent word-label associations, and manual review of the text.

A key component of X-Deep is the incorporation of human feedback. Human involvement is essential for interpreting the nuances revealed by XAI, identifying subtle patterns, and providing qualitative judgments that automated processes might miss. The framework presents XAI explanations to human participants, who then offer feedback to refine and improve the dataset. In this study, the human participants were seven of the authors of the study, all of which are native Arabic speakers with a background in computer science and familiarity with emotion detection tasks. This linguistic and technical expertise was important for interpreting the anomalies within the Arabic dataset and how they related to choices made during the data collection or pre-processing. Optimally, for the X-Deep framework to be most effective, the humans participating in the HitL process should possess relevant subject matter expertise combined with proficiency in the language of the dataset being analyzed. This combination allows for a deeper understanding of context, cultural nuances, and domain-specific issues that are essential for accurately identifying and interpreting data anomalies highlighted by XAI tools.

### Experiments

The chosen dataset^30^ is an Arabic tweets dataset that was aggregated from pre-existing datasets and and newly scrapped tweets and then manually annotated into eight emotion classes: None, anger, joy, sadness, love, sympathy, surprise, and fear. The label “none” was given to tweets that were perceived to contain “no emotion”. Four models were tested: two deep neural networks and two machine learning algorithms. For deep learning classifiers, Gated Recurrent Unit (GRU) and transformer (MARBERT)^31^ models were used. GRU was chosen for being the best performing model for sentiment analysis in related work^32^, while the MARBERT model was chosen for being the state of the art model for Arabic NLP tasks at the time of conducting the experiments^21^. For machine learning classifiers, Naive Bayes (NB) and Logistic Regression (LR) were picked. Those two models were chosen for being the worst case and best case performance respectively in similar NLP tasks^33^.

Four main explorations were conducted: exploring the dataset, comparing the two XAI techniques, generating anomaly patterns, and comparing the four classification models. The first exploration was conducted to determine initial pre-processing; the remaining ones aim to answer the following research questions. **RQ1** How do the performances of LIME and SHAP compare in investigating the nuances of the dataset? **RQ2** How effective is X-Deep with respect to devising a data pre-processing and augmentation plan for refining the current dataset? **RQ3** What insights can XAI provide into the performance of the four classifiers GRU, MARBERT, LR, and NB in the emotion detection task?

The details of each experiment are presented in the following subsections.

#### Exploring the Dataset

An exploratory data analysis phase was conducted to determine the pre-processing techniques suitable to the dataset and the purpose of the experiments to follow. This initial step involved a thorough examination of the dataset characteristics. Specifically, we analyzed the distribution of labels and performed word frequency analyses across the entire corpus. Furthermore, we investigated the prevalence of emojis, both globally and within individual label categories, to identify potential patterns and inform subsequent pre-processing decisions. Observations during this phase included instances of non-standard language usage, such as word repetition (e.g., “no no no”) and character elongation (e.g., “nooooooo”), as well as the presence of non-alphabetic characters. Notably, preliminary analysis highlighted potential challenges, such as the ambiguous labeling criteria associated with the “None” category and the skewed distribution of the most frequent word towards a single label, which could introduce bias in model training. Based on these initial findings, we formulated and evaluated several pre-processing strategies, including various stemming approaches and methods for handling emojis.

**Fig. 2:**
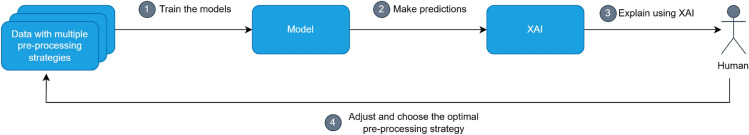
Raw data vs pre-processed experiment pipeline.

Further, transformer models, recognized as the state-of-the-art in NLP, are typically pre-trained on raw text data. To isolate the impact of pre-processing on MARBERT–a model pre-trained on raw text–we compared between two configurations: using common Arabic NLP pre-processing steps (e.g., emoji replacement, stemming, stop-word removal, and text normalization) and another retaining the original, unprocessed text. The evaluation was conducted according to the pipeline in Fig. 2, aimed to determine whether pre-processing enhances the performance for models trained on raw linguistic data, while also assessing its effect on the interpretability of XAI outcomes.

For each pre-processing configuration (including raw data), we independently fine-tuned MARBERT. Classification metrics were then compared, and mismatched predictions were analyzed using XAI. This dual approach evaluated not only predictive accuracy but also the fidelity of explanations, particularly for correct predictions driven by potential spurious correlations.

#### Comparison of the XAI Techniques

**Fig. 3:**
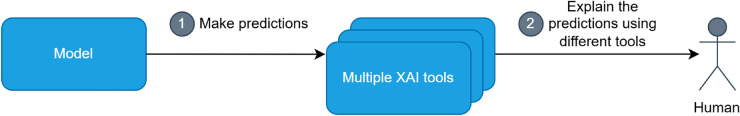
Comparison of the XAI techniques experiment pipeline.

The goal of the experiment is to compare LIME and SHAP for inspecting the nuances of NLP models. The pipeline of the experiment follows Fig. 3. At the outset, we defined preliminary aspects–namely consistency and robustness–that we wanted to evaluate, but remained uncertain about what other behaviors to probe; therefore, we first observed how the tools behaved on a randomly selected subset of the data. The resulting explanations were then scrutinized to identify problematic or particularly interesting anomalies and behavior patterns that required deeper analysis. Subsequently, a targeted selection focused on samples with specific pre-processing or structural properties, such as the presence of repeated words or variations in text length. This targeted approach aimed to determine whether observed characteristics represented isolated occurrences or consistent patterns across similar samples (either by extracting items with matching structures or attributes directly from the dataset, or by manually generating parallel examples). The two XAI techniques were then compared across a number of different aspects.

**Consistency** checks for whether the XAI technique will maintain similar feature weights upon repeated application to the same sample. This is crucial as the framework relies on iterative analysis, and significant variations in weights between runs would compromise the validity of the process. This was tested by running the same sample through both tools multiple times and observing any changes in the weights assigned to words.

**Robustness** assesses the sensitivity of the feature weights to minor perturbations in the input. Testing was done by manually removing words, focusing especially on those with low weights, and observing the resulting effects on the weights of other words.

The handling of **Repeated words** by XAI tools like LIME and SHAP is notably different. By default, LIME employs a Bag-of-Words (BoW) representation, grouping all instances of a repeated word into a single feature and assigning a cumulative weight to the collection as a whole. In contrast, SHAP assigns a distinct weight to each individual occurrence of a repeated term, reflecting its specific position and contextual interactions. This difference makes it crucial to analyze how it impacts the resulting explanations.

**Time complexity** analysis was conducted to provide a heuristic comparison of the computational runtime of the two XAI techniques. This analysis involved examining how each tool operates in both theory and practice.Despite the availability of adequate computing resources, the iterative nature of the analysis highlights the necessity of considering computational efficiency. If the performance of the XAI techniques is comparable, a faster algorithm would be preferred, especially when the model being examined is of higher complexity. These comparisons were conducted for the four classifiers.

#### Generating Anomalies

**Fig. 4:**
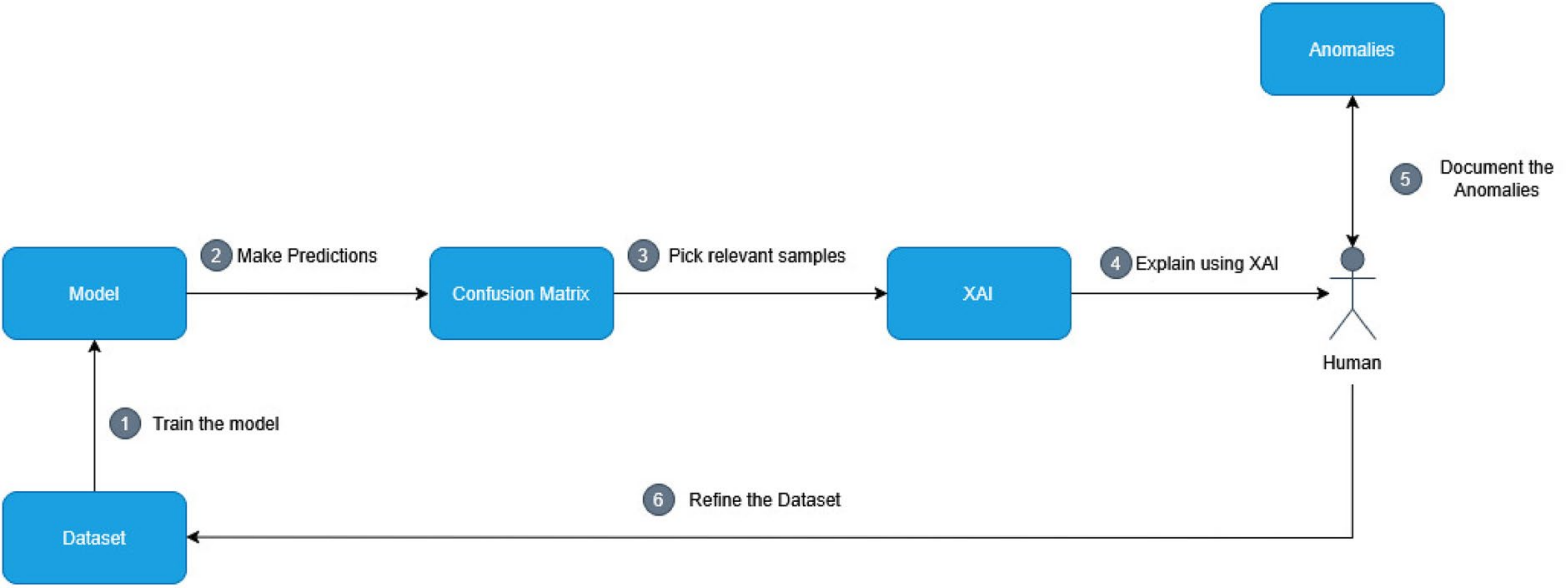
Generating anomalies experiment pipeline.

The goal of this experiment is to systematically identify and document anomaly patterns within the dataset that contribute to classification errors, as shown in Table 1. A suggested list of anomalies was proposed based on initial explorations of the dataset in the previous experiments and prior experience with similar datasets. The pipeline of the experiment, illustrated in Fig. 4, is a multi-stage process designed to set and refine this list and analyze the characteristics and potential impact of the identified anomalies. Note that this experiment focuses exclusively on the identification and validation of these patterns within a dataset, rather than implementing or evaluating specific corrections for the identified anomalies.The framework proceeds through the following stages: **Initial Identification and Inspection** Utilizing confusion matrices generated from the models’ predictions, we selected samples that were incorrectly classified across all target labels. We placed particular focus on labels with a high frequency of misclassifications, labels that were consistently confused with specific other labels, and samples misclassified by more than one model. This broad selection strategy ensured a comprehensive pool of instances for subsequent analysis. The sample-selection stage was also guided by the list of potential anomalies in Table 1. All selected samples were then subjected to in-depth inspection using the XAI tools across all classifiers. Our goal was to understand the specific features or patterns within each instance that contributed to the classification error, thereby characterizing the anomalies present in the data. **XAI-Guided Anomaly Hypothesis Testing:** Each selected sample was analyzed using XAI tools, namely LIME and SHAP. By examining **feature attributions** across similar samples and classifiers, we pinpointed the specific linguistic or structural elements that caused each misclassification. Whenever a recurring pattern emerged–such as problems related to a specific word or dialect, it was designated as an anomaly. Hypotheses that lacked sufficient empirical support were set aside for subsequent review. **Cross-Classifier Sensitivity Analysis** For each newly defined anomaly, we assessed whether other classifiers in our suite exhibited similar error patterns. This step revealed model-specific blind spots and determined whether the anomaly was inherent to the data or specific to a particular model. **Iterative Refinement & Validation** To refine each anomaly’s definition and confirm its impact, we conducted two additional **HitL** passes. In the second iteration, borderline cases were reviewed by either minimally editing existing examples or crafting synthetic ones that embodied the anomaly; we then observed whether removing the anomalous feature would flip the classification outcome. A third pass served purely to validate consistency: every anomaly had to reliably reproduce the failure mode across multiple samples and classifiers. In total, we performed three annotation cycles–identification, refinement, and validation–to converge on a robust, actionable anomaly catalog.

**Table 1:** The list of Anomalies.

Anomaly	Explanation	Arabic example	Translation
Specific words	Popular words that might result in spurious correlations (e.g., hashtags used to scrap the dataset)	اﻻولمبياد	Olympics
Context-sensitive words	Some words used can convey certain emotions but due to the context they end up having a different meaning	يوجعني قلبي وانا خايفه يتركني وانا احبه مجرد ناس عابرين	My heart aches and I’m afraid he’ll leave me, even though I love him. They’re just passing people.
Incorrect labels	Labeled example with an emotion that does not suit that example	أيه الالفاظ المقرفه دي	What are these disgusting words
Diversity of dialects	Sentences in dialects from countries other than Egypt (ex: Tunisia, Saudi Arabia, ...) could be a source of confusion	قول لصالح محبينك من صلاله ونحنا معكم وشدو الحيل زد رصيدك	Tell Saleh that his fans from Salalah are with him, and they are cheering him on. Keep up the good work, and increase your score
Short statements	Statements that becomes shorter than five words after pre-processing may not have enough context to infer an emotion	الجوع ملحد	Hunger knows no religion
Long statements	Statements longer than twenty words after pre-processing may cause confusion with some models	جنود بريطانيون يرمون اوسمتهم امام مبني الحكومه لا يمكننا ان نجني السلام ونحن نقتل مدنيين بحجه داعش وعلي بريطانيا بالانسحاب من التحالف الدولي	In front of the government building, we cannot seek peace while we are killing civilians under the pretext of ISIS, and Britain should withdraw from the international coalition.
Mixed feelings	Examples that convey more than one emotion	ليتنا ماعرفنا بعض ولا عشقنا ،! ليتني غضيت البصر ولا شفتك .. ليت العمر وقف يالغلا ولا تصادقنا ،، ليت مابه حب بهالدنيا وماحبيتًك	We didn’t know each other, nor did we love. I wish I had lowered my gaze and not seen you. I wish time had stopped, my love, and we hadn’t become friends. I wish there was no love in this world and I hadn’t loved you.
Repeated emojis/words	Examples that have a specific word or emoji repeated multiple times may force the model to make a different prediction	مصر في المركز الخامس في اﻻولمبياد (: (: (:	Egypt is in fifth place in the Olympics :) :) :)

#### Comparison of the Models

**Fig. 5:**
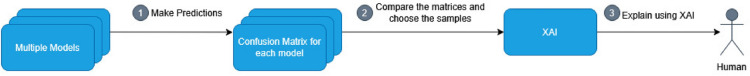
Comparison of the models experiment pipeline.

The goal of this experiment is to gain a better understanding of the particularities of different classification models with the Arabic emotion detection task in general by examining how the XAI techniques justify their predictions. For instance, even when all classifiers predict the same label for a specific sample, the explanations can differ significantly, let alone in cases where models predict different labels and the corresponding explanations diverge. The pipeline of the experiment follows Fig. 5. Samples where all models achieved correct predictions, where all models made incorrect predictions, and where only a single model predicted correctly or incorrectly were selected for explanation. The rationale behind this selection was to identify the unique behavioral characteristics of each model under identical circumstances. These samples were then analyzed using both LIME and SHAP across all classifiers to identify potential trends in model behavior, such as whether two models exhibit similar explanatory patterns or if their behaviors are entirely distinct. Throughout the iterative analysis, observed trends were either confirmed, refuted, or temporarily noted for further investigation based on the consistency of the findings across one or multiple classifiers.

### Implementation Details

All of the experiments were run using an NVIDIA P100 GPU to accelerate training. The dataset was split into 70% for training, 15% for validation, and 15% for testing, and all models were trained and evaluated on the same splits. Furthermore, a fixed checkpoint was used for testing across all experiments to ensure consistency. Hyperparameter optimization was performed for each model to maximize the validation F1-score.

#### Naive Bayes

The Gaussian Naive Bayes implementation was used with variance smoothing set to $1\times 10^{-6}$. This configuration was evaluated both alone and in combination with Linear Discriminant Analysis (LDA) using 44 components. The average inference time per sample was 0.0009 s for both configurations.

#### Logistic Regression

The sklearn LogisticRegression model was used with the “lbfgs” solver and the One-vs-Rest strategy. The model was trained on 86-dimensional embeddings with L2 regularization (inverse regularization strength $C=1$). The model took around 41 s to converge during training and the average inference time per sample was 0.0005 s.

#### GRU

The GRU model comprised of three layers with 128, 64, and 32 units, respectively, interleaved with 20% dropout. The token sequences were embedded into 86-dimensional vectors and processed using gated recurrences. Optimization was performed using Adam (learning rate $6.5\times 10^{-3}$). Training was capped at 100 epochs with early stopping (patience of 10 epochs and restoration of best weights) and mini-batches of size 64. The model converged on average after 11 epochs, requiring approximately 30 s. The average inference time per sample was 0.026 s

#### MARBERT

The pre-trained MARBERTv2 model was used as a base model and fine-tuned for two epochs on the dataset. A batch size of 16 and a learning rate of $3.45\textrm{e}{-5}$ with a weight decay of 0.0156 were used. The model training took around 189.5 s for two epochs. The average inference time per sample was 0.140 s.

## Experimental Results

This section presents the results of the four sets of experiments described in Section “Approach”.

### Exploring the Dataset

**Fig. 6:**
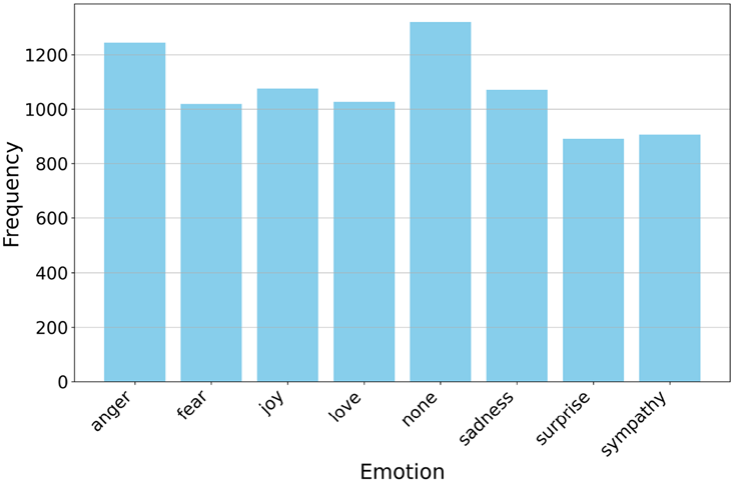
Emotion label distribution from the dataset(anger, fear, joy, love, none, sadness, surprise, sympathy).

**Fig. 7:**
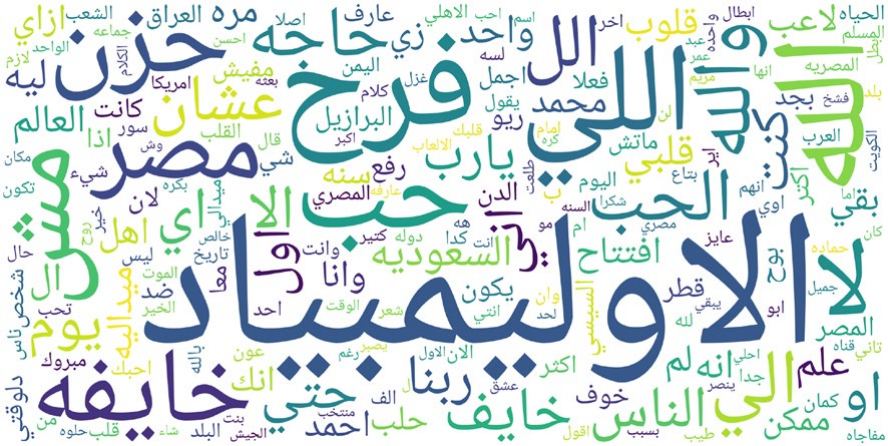
Word cloud from the processed dataset where the most common words are (Olympics/الاولمبياد), (Egypt/مصر), (God/الله) and emotional words like (happiness/فرح), (love/حب), (sadness/حزن) and (fear/خايفه), alongside linking words like (اللي) and negation words (مش).

An exploratory data analysis phase was conducted to characterize the corpus and help decide on an appropriate pre-processing strategy. The distribution of the emotion labels in the dataset was found to be relatively balanced, without significant disparities, as illustrated in Fig. 6. Word clouds were utilized to gain insights into the textual content, as shown in Fig. 7, where the word “الاولمبياد” (Olympics in Arabic) is observed to be the most frequently occurring term in the dataset and appears predominantly within the *none* class. This observation suggests a potential for spurious correlations during model training. Furthermore, while the *none* class was formally defined as encompassing “sentences that carry no discernible emotion,” in practice, annotators appear to have included instances that did not clearly align with the other defined emotion categories. For example, sarcastic praise of a rival team’s performance (e.g., mock acclaim after a humiliating loss) or disdain for a sports team’s uniform design were frequently labeled as ‘none’, despite conveying clear emotional undertones misaligned with the predefined categories.

**Fig. 8:**
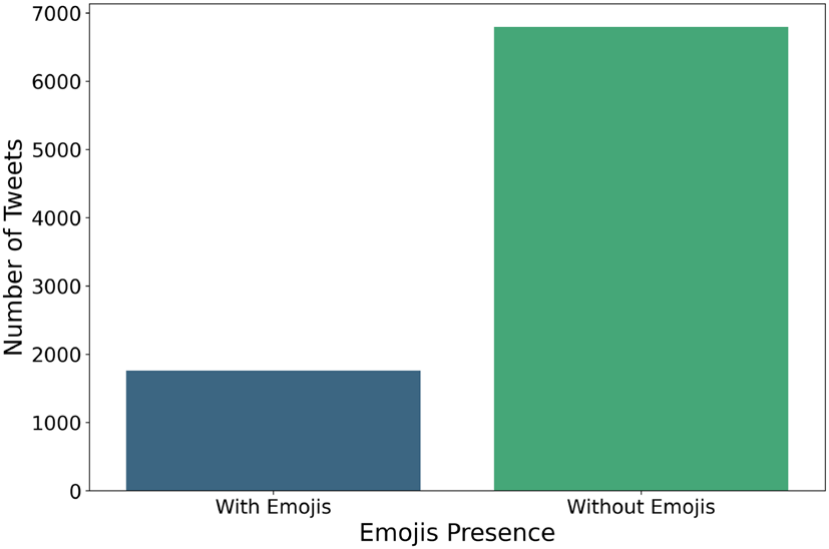
Comparison of the number of tweets with and without emojis.

**Fig. 9:**
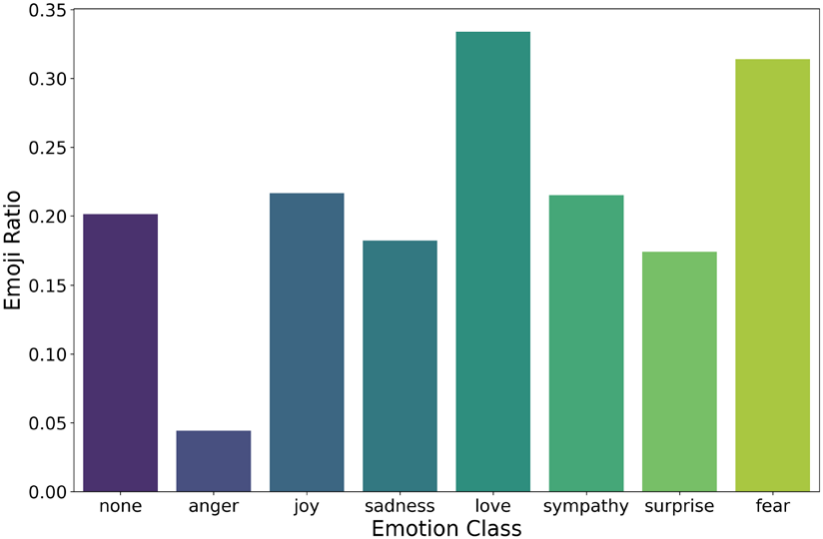
Ratio of emoji usage across emotion classes (e.g., joy, fear, love).

In the dataset, most tweets do not include emojis as shown in Fig. 8 with an emoji use ratio of 18%. However, there’s a clear relation between emoji usage and labels as shown in Fig. 9. For instance, ‘anger’ tweets have significantly lower emoji usage, while ‘love’ and ‘fear’ exhibit higher emoji usage (34% for ‘love’). Surprisingly, the class ‘None,’ representing a lack of emotion, has around 20% emoji usage. This suggests that emojis should remain as part of the text to hold the intended meaning.

Based on the exploratory analysis, several pre-processing techniques were employed. Initially, emojis are mapped to certain emotion categories, with each emoji being handled as a separate word. Emojis that fail to effectively convey emotions are removed. The text is then normalized by eliminating non-Arabic characters, numerals, and punctuation marks.

Stop words are filtered out. A mild stemmer ISRI stemmer from the NLTK library^34^ was used to balance readability, which would be important to interpret XAI results, with word reduction to their most basic forms. MARBERT was used to generate word embedding by obtaining the output for each word from the final layer of the model. For models that need their input to have constant dimensions like LR and NB, the embedding of the whole sentence was calculated by taking the average of the word embeddings. Table 2 summarizes both the classification results for all four models on the pre-processed data and the comparison of MARBERT’s performance on raw versus pre-processed data.

**Table 2:** Classification metrics: (a) All models on pre-processed data; (b) MARBERT raw Vs pre-processed.

Metric	NB	LR	GRU	MARBERT
(a) All models with data pre-processing				
Accuracy	53%	71%	71%	76%
Precision	53%	72%	72%	76%
Recall	52%	72%	71%	76%
F1-Score	53%	71%	70%	76%

Metric	Raw	Pre-processed		
(b) MARBERT: raw Vs pre-processed				
Accuracy	80%	76%		
Avg F1-Score	80%	76%		

MARBERT exhibited the best performance, significantly outperforming GRU, LR, and NB; these findings are consistent with previous studies^21,33^. Moreover, MARBERT without text pre-processing achieved a 4% higher accuracy and average F1-score compared to its performance on pre-processed data, as shown in Table 2(b).

Beyond the observed accuracy improvements, a detailed review of 200 misclassified samples, analyzed using XAI across different preprocessing methods, revealed valuable insights. Utilizing raw data increases the model’s susceptibility to noise and data variations. A case in point is the phenomenon of exaggeration in Arabic words, where the repetition of characters serves to amplify the meaning, as exemplified by ‘حرااااااام’and ‘حرام’ (both denoting unfairness but with differing degrees of emphasis). This variation can lead to the assignment of different weights and potentially different label associations for each orthographic variant, thereby diminishing the model’s predictability and explainability. Interestingly, the analysis additionally revealed instances where stop words exhibited unexpectedly high feature weights. Employing pre-processing techniques to normalize such variations, including the removal of repeated letters, effectively mitigates this issue.

Further, both LIME and SHAP can be affected when working with raw data. LIME can’t interpret the punctuation marks or the emojis which would lead to inaccurate explanations when sentences include them, this was highlighted in multiple examples, where the question marks (?) had the most significant weight on the prediction when using SHAP, whereas LIME assigned them no weight because it cannot process them. The same issue applies to emojis and other punctuation marks.” Furthermore, the explainability of raw data gives weights to stop words. Exclamation marks (!), question marks (?), emojis, and emoticons seem to improve the model’s performance as well, but as mentioned before they don’t help with explainability. In conclusion, raw data achieved higher accuracies but poorer interpretability and model stability. Since the study focuses on explainability, pre-processed data was used with all models.

### Comparing XAI Techniques

The goal of this experiment was to compare the two XAI techniques, LIME and SHAP, with respect to their performance consistency, robustness to changes in data, behavior with repeated words, and time complexity.

#### Consistency

SHAP has demonstrated greater consistency in its output compared to LIME, which may be attributed to the inherent randomness of the latter. Repeated explanations of the same data using LIME yielded varying weights with each iteration, although the general interpretation remained consistent. The most significant word always persisted, and the probabilities of each label appeared stable. The criticality of the case arises when numerical precision in the weights is required. For example, rerunning the same sample through LIME multiple times resulted in fluctuations of approximately 0.01 to 0.02 in the assigned word weights. This variability was most noticeable in samples where prediction probabilities for multiple labels were very close. Such inconsistency presents a significant challenge when multiple words already possess similar weights, as it undermines the ability of the tool to perform precise fine-grained analysis in these situations.

#### Robustness

When words with minor weights are removed, ideally, this should not significantly impact the provided explanation. In our observations, LIME’s explanations remained largely unaffected by such removals. However, in SHAP explanations, we noted instances where a word that originally had no weight in the sentence gained a new, non-zero weight after the removal, even though the removed words themselves had insignificant weights. This phenomenon occurs because SHAP does not calculate each word’s contribution in isolation but rather considers the entire input as a whole. It is important to note that, in these cases, the overall prediction result did not change. The significance of this observation is that SHAP’s word weights should be interpreted as relative contributions within the context of the input.

#### Behavior with Repeated Words

Repeated words in a sample are usually due to people repeating a certain word (e.g., “love love love”) or an emoji (replaced with a word representing the emotion during pre-processing). This repetition often emphasizes an emotion. However, how XAI tools handle repetitions reveals limitations in providing useful text interpretations.

For instance, LIME’s approach of aggregating repeated words by assigning a single cumulative value to all instances, can obscure how different occurrences of a word contribute to the output in varying contexts or positions. On the other hand, SHAP assigns individual weights to each repetition, theoretically preserving positional information. However, this creates issues–for example, if a word is repeated ten times consecutively, SHAP generates ten distinct weights, causing visual clutter for the user. These scores often lack meaningful differences (e.g., the 6th repetition’s contribution rarely differs from the 5th), undermining their interpretive value.

These shortcomings highlight the need to balance granularity (for detail) and abstraction (for clarity), as neither tool achieves this inherently. To address this, pre-processing could limit repetitions to a threshold (e.g., three instances). Additionally, LIME’s default BOW tokenization could be replaced with context-aware alternatives to better capture positional relationships between repeated words.

#### Time Complexity

Analysis of the theoretical runtimes of the XAI tools shows that SHAP’s Partition Explainer, which is primarily used for text explanations, runs in $O(d^2 \cdot t)$ time, where $d$ is the number of input features and $t$ is the model’s end-to-end runtime. LIME also scales roughly quadratically for moderate values of $d$, but its overall cost is multiplied by the number of perturbations $N$ (typically in the hundreds or thousands), resulting in $O(N \cdot d^2 \cdot t)$ runtime. In practice, this extra factor makes LIME substantially slower—especially when explaining more complex models with computationally expensive components.

### Generating Anomalies

This experiment sought to identify distinctive anomaly patterns in the dataset, following the pipeline described in Section “Experiments”. The most striking trends and correlations identified are summarized here.

1. The word “الاوليمبياد” (Olympics) tends to have a spurious correlation with the label ‘none’.
2. The word “الله” (God) and any kind of prayers-related words tend to be classified as ‘sympathy’ disregarding the true label (potential bias).
3. Poetry or songs tend to be classified as Love disregarding the true label (potential bias).
4. Religious talk tends to be classified as anger disregarding the true label (potential bias).
5. Politics and public characters tend to be classified as anger disregarding the true label (potential bias).
6. Tweets exhibiting mixed feelings are -predictably- a problem with all classifiers.
7. Different dialects often result in misclassifications.
8. Some samples can’t be appropriately classified as one of the eight classes such as disgust and sarcasm.
9. The abuse of emojis is prevalent in social media and thus in this dataset too, resulting in some misclassifications.
10. Many times, the classifiers’ predictions and explanations were more accurate than the true data label, especially using the MARBERT model.

These trends were observed and validated across all classifiers. That doesn’t mean that each example in those trends holds true for all classifiers all the time, but it means that in the general sense it holds true for the classifiers most of the time. Some of these anomalies are expanded upon in the paragraphs below.

XAI analysis uncovered a spurious correlation in the dataset involving the word اﻻوليمبياد (Olympics). Since a subset of the dataset was collected during Olympic events, the term appeared with unusually high frequency. This skewed its statistical correlation with the label ‘none’, biased the model to disproportionately classify tweets containing “Olympics” as ‘none’. To confirm this observation, the word “Olympics” was replaced in misclassified tweets with synonyms or generic terms that hold a similar meaning, which resulted in correct label predictions for the majority of cases.

Another observed pattern in misclassified examples stemmed from contextual misunderstandings. For instance, tweets containing Islamic duā (prayers) expressing sympathy for a sick person were more likely to be misclassified as ‘love’. Similarly, political or religious topics were frequently misclassified as ‘anger’, regardless of the tweet’s content. These errors suggest that biases can manifest on multiple levels, not only through repeated words but also through contextual associations. For example, if certain topics (e.g., politics, religion, Poetry) have a high frequency of a specific label (e.g., ‘anger’, ’love’) in the training data, the model may overgeneralize, conflating domain-specific language with emotion. This underscores the need for datasets to prioritize diversity, not just in topical or lexical coverage but also in the distribution of labels associated with those topics.

The model exhibited higher misclassification rates for samples written in dialects other than Egyptian (the primary dialect in the training dataset). For example, a Gulf region dialect phrase ما عُمري (“I never”)–a grammatical construct uncommon in Egyptian dialects–was incorrectly labeled as ‘anger’ despite its neutral intent. This suggests the model struggles with dialect-specific syntactic and lexical variations, misinterpreting unfamiliar constructs as sentiment-bearing. Such errors highlight the need for dialect-aware training data.

Short statements were observed to be more prone to misclassification. For example, replacing the word مُرعب (horrific) with خوف (fear) in a short sentence caused the predicted label to shift from ‘sympathy’ (incorrect) to ‘fear’ (correct). This occurred despite both words conveying semantically equivalent meanings, highlighting a concerning level of instability in the model’s sensitivity to lexical substitutions. Such instability persisted across various labels and contexts in short texts, underscoring a broader problem: minor wording changes disproportionately affect predictions, even when the semantic intent remains unchanged.

On the other hand, long statements can sometimes confuse some models because the sentiment in a very long sentence is not always constant across the whole sentence, which would lead simpler models to misclassify them.

### Comparison of the Models

In order to better understand the behaviour of the classifiers, the aim of this experiment was to use XAI to identify patterns peculiar to the classifiers in dealing with this dataset, if any.

#### Naive Bayes

Naive Bayes(NB) treats each feature independently; it gives weights to each word independently, which makes it difficult to infer the label from the whole context. This becomes apparent when there are repeated words in the sample being processed, as it gives a considerable weight to each occurrence of the word, so in cases of repeated words or emojis, these words drive the prediction of the sentence into a certain label. For example, repeated occurrences of a word like حُب (love) are treated independently. In practice, this leads to a situation whereby a sentence with multiple repetitions of حُب is misclassified as ‘love’, when the true sentiment is ‘happiness’.

The issue arises because NB overweights term frequency: each repetition of حُب increases the probability for ‘love’, drowning out other contextual signals. When repetitions are removed (e.g., reducing “حُب حُب حُب” to a single حُب), NB’s prediction shifts to the correct label, demonstrating its reliance on surface-level lexical patterns rather than semantic understanding.

**Fig. 10:**
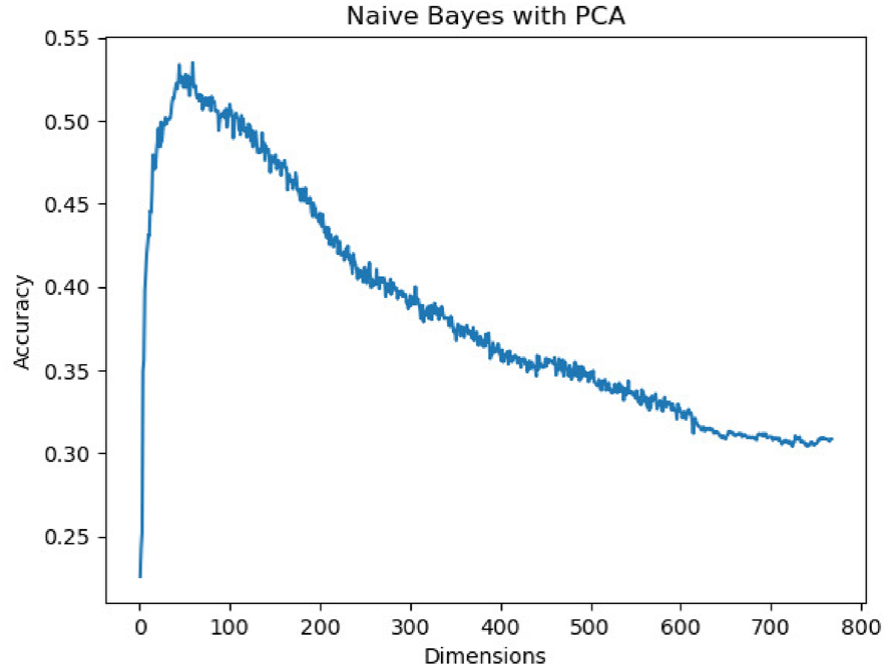
The effect of PCA on Naive Bayes model.

It was hypothesized that dimensionality reduction would improve the performance of NB by summarizing the features input into the model and reducing the severity of the independence problem. Applying Principal Component Analysis (PCA) enhanced the performance going from 49.5% test accuracy to 53.4% (see Fig. 10). Using Linear Discriminant Analysis (LDA) has further enhanced the performance to 70%. In contrast to more complex models like MARBERT and GRU, NB with LDA has a very small model size, a quick prediction time, and very few hyperparameters. Despite this, NB with LDA achieves results that are very competitive with these models, making it a plausible option for real-time applications or mobile applications with limited resources. Although this particular observation is not a result of applying XAI, the techniques, however, made it clear why it is needed. This observation supports the employment of weaker models with quick inference times because they can be improved and adjusted to produce competitive results. Dimensionality reduction negatively affects the other ML model (LR) as it results in feature loss, therefore less information for the models to train, bearing in mind that these models did not suffer from the independence problem; so there is no problem to solve here in the first place.

#### Logistic Regression

Logistic Regression showed a performance level between NB and GRU, achieving test accuracy around 71%. It handled the features better than NB, but lacked the capacity to model sequence dependencies or complex contextual cues. XAI tools revealed that LR focused on high-weighted keywords and performed well when strong lexical indicators of emotion were present. However, it struggled with subtle expressions or when emotion was implied indirectly. Unlike GRU or MARBERT, it didn’t display any unique behavior in terms of attention patterns or feature reliance. It served as a useful baseline but did not demonstrate characteristics that made it particularly suited to this task beyond its simplicity and interpretability.

#### GRU

GRU achieved reasonable performance (approximately 71% test accuracy), comparable to Logistic Regression but trailing MARBERT, and its contextual modeling outperformed NB yet fell short of transformer-based approaches. However, XAI analyses revealed that GRU often placed disproportionate focus on named entities and titles rather than on the actual emotion-bearing content. For instance, in the tweet “احساس خالد عبدالرحمن لا يوصف” (“Khaled Abdelrahman’s feeling cannot be described”), the model assigned the highest attention weight to “خالد عبدالرحمن” (“Khaled Abdelrahman”), which led to a misclassification. In another example describing a heroic act by a “ملازم” (“lieutenant”), the model similarly focused mainly on the title rather than the content of the tweet. These findings demonstrate that, although the GRU captures sentence-level structure better than simpler models, it remains vulnerable to dataset biases and overfitting to specific entities.

#### MARBERT

MARBERT consistently outperformed the other models, achieving the highest test accuracy (76%). It demonstrated superior contextual understanding, effectively handling nuances of Arabic sentence structure and idiomatic expressions. Through explainability analysis, MARBERT was observed to sometimes “correct” mislabeled examples, for instance, predicting “surprise” in cases where the true label was “fear”, but where surprise was arguably more appropriate based on the tweet’s content . This suggests that MARBERT can serve not only as a classifier but also as a tool for dataset auditing, flagging potential labeling inconsistencies for human review.

Moreover, MARBERT proved less sensitive to surface-level features like repetition compared to Naive Bayes (NB). For example, in samples with emphatic repetitions–such as “حُب حُب حُب” (love)–MARBERT interpreted these as stylistic markers of emotional intensity (e.g., signaling ‘happiness’) rather than literal evidence for the ‘love’ label. This contrasts sharply with NB, which misclassified such repetitions.

However, MARBERT’s advanced contextual awareness did not protect it from subtler biases rooted in dataset patterns. For instance, the spurious correlation between the word اﻻوليمبياد (Olympics) and the label ‘none’–a result of topic-skewed training data–persisted in MARBERT’s predictions. Unlike NB, which tends to overfit by focusing on repeated words or phrases, MARBERT shows a different kind of bias driven by context. Its ability to detect thematic patterns sometimes leads to misclassification. For instance, tweets about the Olympics were frequently labeled as ‘none’, even when they clearly expressed sentiment, because MARBERT learned to associate the overall topic (Olympics) with neutral content, based on its high occurrence in ‘none’-labeled tweets within the dataset.

## Discussion

This research addressed three key questions through a case study. First, in response to **How do the performances of LIME and SHAP compare in investigating dataset nuances?**, the findings showed both techniques had comparable performance, although LIME demonstrated unique effectiveness in handling punctuation marks and repeated words, suggesting these elements need special pre-processing. SHAP, on the other hand, was more time-efficient. Both LIME and SHAP exhibited sensitivities: LIME was sensitive to re-running the same data, while SHAP was sensitive to slight input changes. This impacts result interpretability and indicates that the choice of pre-processing techniques can influence the results.

For the second question **How effectivfe is X-Deep in refining a dataset through data pre-processing and augmentation?**, X-Deep has effectively helped in identifying anomaly patterns that could guide data augmentation strategies. For instance, the framework showed that MARBERT consistently outperformed the other classifiers, assigning labels that aligned more closely with each sample’s true sentiment sometimes even more than the original annotations did. which could help in validating dataset labels. Further insights suggested handling short sentences differently by augmenting them with filler words, while longer sentences, often expressing more than one emotion, could be removed. Word repetition, particularly due to emoji handling, was handled inconsistently across classifiers, with Naive Bayes being more sensitive to repetition than Logistic Regression or Transformer-based models. We will expand more on the anomaly mitigation strategies in Section “Mitigation strategies”. While these findings are based on Arabic tweets, many of the observed patterns, such as classifier sensitivity to word repetition or sentence length, are not unique to Arabic and are expected to occur in other languages as well. At the same time, some patterns were specific to Arabic, such as ambiguity caused by different dialects and challenges related to complex morphology. This suggests that while language-specific behavior may influence identified anomalies, some of the identified issues are language-agnostic. Other languages may present their own unique patterns that, once identified, could be integrated into the workflow to further support the framework’s effectiveness.

Regarding the third question **What insights can XAI offer into the performance of classifiers (GRU, MARBERT, LR, NB)?**, XAI techniques provided valuable insights. GRU struggled with compound Arabic names, MARBERT excelled at correcting label errors, and dimension reduction significantly improved Naive Bayes performance. This highlighted the importance of examining misclassifications for error analysis. It is additionally advisable not to depend solely on accuracy to compare the performance of classifiers. Overall, the comprehensive analysis, enabled by XAI, emphasized the significance of documenting these particularities to better inform the choice and use of classifiers and improve model performance in the case of NB.

### Mitigation Strategies

By leveraging the insights from the framework and the list of identified anomalies, targeted strategies can be developed to address these issues, while demonstrating how systematic anomaly detection can strengthen overall model robustness and performance. For example, spurious correlations were detected between certain words (e.g., اﻻوليمبياد, “Olympics”) and specific labels (e.g., “none”), and political content was often linked with anger. To combat the issue of spurious correlations, the data collection process should account for biases introduced by collecting data on time-limited events (e.g., high-profile sporting, political, or social occurrences). If collecting such data is needed, measures should be taken to ensure that no event or word is overrepresented in the dataset. This could be done by reducing the number of samples that contribute to the bias or replacing words that are overrepresented by appropriate synonyms. Proactively identifying and mitigating these correlations through anomaly detection not only reduces overfitting, but also improves the model’s ability to generalize to unseen data, as it minimizes reliance on transient or context-specific patterns.

The framework also reveals weaknesses in the annotation process. The presence of mislabeled samples highlights the need to increase both the number of annotators and their demographic diversity to increase the likelihood that the labels reflect the true underlying classes. Additionally, the annotation scheme itself may require refinement, as the presence of samples that did not fit any of the pre-existing classes lead to them being labeled and annotated as ’none’, which points to a need for more classes or a more nuanced scheme, such as a multiclass framework or a continuous scale, to capture the full spectrum of emotions in the data. This, in turn, would enhance the model’s semantic understanding ability and reduces ambiguity in training data, directly improving the robustness of the model.

Dialect-related misclassifications revealed by the framework highlight another critical area to be aware of. This could be addressed by restricting the data sourcing to the geographic region and dialects for which the model is intended, which was done to some extent with this dataset but was not enough to limit the appearance of this issue, pointing to the need for more exploration of the dialectical impact on model performance. Finally, the framework exposes vulnerabilities in handling specific text structures, like short sentences or word repetition. To improve robustness, models should be rigorously tested for consistency when processing such inputs, and pre-processing steps such as imposing a minimum word count to ensure adequate context or filtering out excessive repetitions should be considered to further enhance prediction reliability.

The integration of error analysis and anomaly detection into model development pipelines offers two key advantages. First, it acts as a diagnostic tool, uncovering subtle biases and inconsistencies that traditional evaluation metrics might overlook. Second, it facilitates proactive mitigation, enabling iterative improvements to data quality, annotation protocols, and pre-processing pipelines. By systematically addressing anomalies, models become less susceptible to edge cases and distribution shifts, ensuring performance remains stable across diverse inputs.

### X-Deep Framework Utility

The X-Deep framework demonstrated the value of XAI in error analysis, revealing root causes of misclassifications and identifying biases and spurious correlations in the data. This process is essential in DCAI approaches for refining datasets and guiding future data collection. For example, a spurious correlation was discovered between specific terms used to collect the data and the ’none’ class, and biases between religious and poetry texts were identified. XAI’s systematic approach, combined with human input, revealed subtle patterns that might otherwise go unnoticed, especially in larger datasets. Although only a small number of such anomalies were detected, their presence in a relatively small dataset is alarming, suggesting that more might emerge as the dataset grows.

The iterative error analysis involved multiple classifiers and XAI techniques, confirming the consistency of detected patterns across classifiers. Both LIME and SHAP performed similarly, indicating that one technique might be sufficient for such investigations. In this case study, the inclusion of various machine learning (ML) and deep learning (DL) classifiers ensured comprehensiveness, though DL models like MARBERT consistently yielded better results, suggesting that one or two DL techniques might suffice in future work.

One significant takeaway from this study is the need to document anomaly patterns, as these patterns will simplify future data cleaning and refinement for similar datasets. This could provide AI developers with starting points to investigate. While anomaly detection is a standard practice for data engineers, its importance is often overlooked in the data-centric approach. Our framework sheds light on the value of anomaly detection in guiding further data collection and dataset refinement. Moreover, the study underscores the importance of human involvement in the analysis process. While aspects of the framework could be automated, human intuition remains essential in identifying subtle patterns, as demonstrated in our case study.

The state-of-the-art performance in Arabic emotion detection is relatively average, and conventional wisdom suggests simply increasing dataset size. However, the findings of this study show that systematic inspection of errors using XAI can significantly improve data collection and pre-processing strategies. By addressing issues such as spurious correlations and biases, this research provides insights into the best practices for refining Arabic emotion datasets, an area that, to the best of our knowledge, is still under-served in NLP literature.

## Conclusion and Future Work

This research introduced a DCAI framework that utilizes XAI tools and human sensibility in the development cycle of emotion detection models. This framework aims to improve the development cycle by helping developers better understand model weakness, data biases, and XAI interpretations that stem from the collected data. Multi-class emotion analysis in Arabic was used as a case study to demonstrate how this framework operated. Naive Bayes, Logistic Regression, GRU, and MARBERT based models were developed and investigated using X-Deep. Certain weaknesses and biases in the dataset were identified leveraging LIME and SHAP XAI techniques. Comparing different XAI tools demonstrated their differences in handling Arabic NLP data. The comprehensive analysis using X-Deep framework has led to the formulation of multiple mitigation strategies that could be acted upon to address the issues uncovered in the dataset. While the proposed framework yielded promising results, we acknowledge that the study is limited to Arabic-language tweets from a single domain. This limitation may affect the generalization of the findings to other languages or application areas. Future work should extend the evaluation to datasets in other languages and broader tasks to assess the framework’s adaptability and robustness, and should systematically test the mitigation strategies outlined in Section “Mitigation strategies”, evaluating their efficacy in improving model robustness, generalization, and fairness across diverse datasets. Moreover, it would be interesting to test the framework in computer vision data using XAI tools like Grad-Cam^35^.

## Data Availability

The dataset analysed during the current study are available in the Emotional-Tone repository, https://github.com/amrmalkhatib/Emotional-Tone, with all user-identifying information removed. Only tweet texts are retained and available. We did not perform any additional data collection. Based on commonly accepted academic standards and practices at our institution, the use of this publicly available and anonymized dataset does not require additional ethical approval.
